# Bile Acid Metabolism in Gout Pathogenesis from Gut–Liver–Joint Crosstalk to Therapeutic Opportunities

**DOI:** 10.3390/metabo16070464

**Published:** 2026-07-02

**Authors:** Beiyan Chen, Xin Chen, Jing Li, Shuang Gao, Xuezhu Wang, Jieru Han

**Affiliations:** 1 Graduate School, Heilongjiang University of Chinese Medicine, Harbin 150040, China; chenbeiyanabc@163.com (B.C.); gaoshuangacc@163.com (S.G.); 2 First Clinical Medical College, Heilongjiang University of Chinese Medicine, Harbin 150040, China; radinen@outlook.com (X.C.); wxzdyx2006@163.com (X.W.); 3 Second Clinical Medical College, Heilongjiang University of Chinese Medicine, Harbin 150040, China; 18762035658@163.com; 4 School of Basic Medical Sciences, Heilongjiang University of Chinese Medicine, Harbin 150040, China

**Keywords:** bile acids, gout, hyperuricemia, FXR, TGR5, NLRP3 inflammasome, gut microbiota, enterohepatic circulation, PPAR-α, uric acid

## Abstract

Beyond their established role in lipid digestion, bile acids function as key metabolic and immune signaling molecules. This review synthesizes recent advances in bile acid metabolism within the context of gout and hyperuricemia, proposing a gut–liver–joint crosstalk framework. Dysregulated bile acid metabolism—characterized by a reduced total bile acid pool, decreased hydrophobic secondary bile acids, elevated 12α-hydroxy bile acids, and impaired enterohepatic circulation—has been mechanistically linked to both hepatic urate overproduction via the PPAR-α/xanthine oxidase pathway and monosodium urate crystal-induced NLRP3 inflammasome activation, although human causal evidence remains to be established. The nuclear receptor FXR suppresses NLRP3 at the transcriptional level, while the membrane receptor TGR5 acts post-translationally through Cyclic adenosine monophosphate/Protein Kinase A (cAMP/PKA) and Glucagon-like peptide-1 (GLP-1) signaling. Gut microbiota dysbiosis amplifies these abnormalities through a vicious cycle of reduced bile acid signaling, increased intestinal permeability, and systemic endotoxemia. Based on these insights, we summarize five therapeutic strategies: FXR modulators, TGR5 agonists, microbiota-based interventions, natural products, and ursodeoxycholic acid replacement therapy. Future research should prioritize gout-specific preclinical models, clinical trials of TGR5 agonists, standardized microbiota-based therapies, dual-target molecules, and personalized patient stratification based on bile acid profiles.

## 1. Introduction

The classical paradigm of gout pathogenesis follows a pyramid model: hyperuricemia leads to monosodium urate (MSU) crystal formation, which in turn triggers acute inflammatory arthritis. However, several clinical paradoxes challenge this linear view [1]. For instance, only 10–15% of individuals with hyperuricemia eventually develop gout [2], and recurrent flares often persist even after serum urate levels are normalized [3]. These observations suggest that additional factors beyond urate burden—particularly those integrating metabolic, immune, and microbiome signals—are required to fully explain gout development and progression [4].

Over the past two decades, bile acid research has undergone a paradigm shift. The discovery of the farnesoid X receptor (FXR) and the G protein-coupled bile acid receptor 1 (TGR5) has redefined bile acids as genuine integrative signals linking metabolism and immunity [5,6]. As summarized in a key Nature-series review, dysregulated bile acid metabolism is now recognized as a common driver of metabolic and inflammatory diseases, ranging from non-alcoholic steatohepatitis to inflammatory bowel disease [7].

The period 2024–2025 has witnessed landmark advances at the intersection of bile acid biology and gout pathogenesis. Metabolomic studies have revealed distinct bile acid profiles in gout patients compared with healthy controls [8,9]. Experimental work has demonstrated that activation of FXR or TGR5 potently suppresses MSU crystal-induced LRR and PYD domains-containing protein 3 (NLRP3) inflammasome activation [10,11]. Moreover, clinical cohort data have begun to unravel the gut microbiota–bile acid axis as a critical modulator in the transition from asymptomatic hyperuricemia to frank gout [12,13]. These findings collectively position bile acid signaling as a previously unrecognized player in gout [14].

To address these questions, we examine how disturbed bile acid metabolism contributes to gout pathogenesis via three interconnected levels—hepatic urate production, gut microbiota modulation, and immune regulation of inflammation. We then evaluate the potential of FXR and TGR5 as druggable targets [15]. Finally, we discuss the translational pathway from preclinical findings to clinical practice. Through the lens of gut–liver–joint crosstalk, this review aims to offer a unifying framework and identify actionable opportunities for targeting bile acid signaling in gout [16].

## 2. A Primer on Bile Acid Metabolism

### 2.1. Chemical Classification and Metabolic Pathways of Bile Acids

Bile acids are key end products of cholesterol metabolism [17]. Their biosynthesis occurs through two complementary enzymatic routes: the classical (neutral) pathway and the alternative (acidic) pathway, initiated by cholesterol 7α-hydroxylase (CYP7A1) and sterol 27-hydroxylase (CYP27A1), respectively [18]. The classical pathway accounts for approximately 90% of total bile acid production in humans, and its rate-limiting step, CYP7A1, is under stringent negative feedback regulation [19]. Within this pathway, the activity of CYP8B1 (sterol 12α-hydroxylase) determines the product profile: when CYP8B1 is active, cholic acid (CA) is generated; when CYP8B1 is suppressed, chenodeoxycholic acid (CDCA) is generated [20]. CA is the only primary bile acid that possesses a 12α-hydroxy group [21]. This structural feature has gained particular attention in gout research, as 12α-hydroxy bile acids (CA, DCA, and their conjugates) are significantly elevated in hyperuricemic states and have been mechanistically linked to xanthine oxidase activity and urate overproduction [13,14]. The alternative pathway begins in the hepatocyte mitochondria, where CYP27A1 catalyzes hydroxylation at the 27-position of the cholesterol side chain, leading predominantly to CDCA. This pathway becomes particularly important during neonatal development or when the classical pathway is impaired [22].

The CA and CDCA synthesized in the liver are collectively termed primary bile acids [23]. Within hepatocytes, they are conjugated with glycine or taurine to form conjugated bile acids (e.g., glycocholic acid, GCA; taurocholic acid, TCA), which enhances water solubility and detergent capacity in the intestinal lumen [24]. Upon entry into the gut, primary bile acids and their conjugates serve as substrates for the gut microbiota [25]. Specific anaerobic bacteria (e.g., *Clostridium* species) catalyze a 7α-dehydroxylation reaction, converting CA into deoxycholic acid (DCA) and CDCA into lithocholic acid (LCA); these are the secondary bile acids [26]. In addition, the microbiota can convert CDCA into ursodeoxycholic acid (UDCA), a hydrophilic, cytoprotective secondary bile acid [27].

CYP7A1 regulation and its relevance to gout. CYP7A1 is negatively regulated by bile acids via hepatic FXR/SHP/LRH-1 and intestinal FGF19/FGFR4/FOXO1 pathways. CYP8B1 is suppressed by hydrophobic bile acids (via FTF/HNF4α competition) and thyroid hormone, but induced by fasting via PPAR-α. These distinct mechanisms allow independent regulation of bile acid pool size and composition [19,20,28]. The relevance of this regulatory architecture to gout is twofold: first, FXR activation suppresses CYP7A1 and further contracts the already reduced bile acid pool in gout patients; second, the 12α-hydroxylated bile acid profile (determined by CYP8B1 activity) is directly linked to urate metabolism through PPAR-α/XOD pathway activation.

### 2.2. Enterohepatic Circulation and Systemic Distribution of Bile Acids

The enterohepatic circulation is the core mechanism maintaining bile acid homeostasis [29,30]. It not only ensures efficient reutilization of the limited bile acid pool but also serves as the basis for bile acids to act as systemic signaling molecules [31]. Primary bile acids and their conjugates, synthesized in the liver, are secreted via the bile ducts and stored in the gallbladder [32]. Upon food intake, cholecystokinin stimulates gallbladder contraction, releasing bile into the duodenum [33]. Approximately 95% of bile acids are actively reabsorbed in the terminal ileum via the apical sodium-dependent bile acid transporter (ASBT), returned to the liver through the portal vein, and taken up by hepatocytes via transporters such as the sodium–taurocholate cotransporting polypeptide (NTCP), thereby completing the cycle [34]. The remaining ~5% of unabsorbed bile acids enter the colon, where they are converted into secondary bile acids by the gut microbiota; a fraction of these is passively absorbed back into the portal circulation, while the rest is excreted in feces [35].

ASBT and NTCP function in bile acid recycling. ASBT (SLC10A2), expressed on the apical membrane of terminal ileal enterocytes, mediates sodium-dependent active reabsorption of approximately 95% of conjugated bile acids, enabling efficient recycling of the bile acid pool [34]. Without ASBT, excessive fecal bile acid loss would deplete the circulating pool. NTCP (SLC10A1), localized on the sinusoidal membrane of hepatocytes, is the predominant transporter for sodium-dependent uptake of conjugated bile acids from portal blood [32]. Its high-affinity uptake prevents bile acids from escaping into systemic circulation and ensures efficient hepatic retrieval for re-secretion [35]. Both transporters are downregulated by FXR-mediated feedback to prevent bile acid overload—a regulatory balance that is disrupted in hyperuricemia, contributing to impaired enterohepatic circulation [13].

Bile acid recycling minimizes the need for de novo synthesis through two complementary mechanisms. First, the enterohepatic circulation reabsorbs approximately 90–95% of secreted bile acids via ASBT in the terminal ileum, physically minimizing the amount that must be newly synthesized to maintain the circulating pool [34]. Second, the recycled bile acids themselves serve as signaling molecules that activate FXR, which in turn suppresses CYP7A1 transcription through the hepatic SHP and intestinal FGF19 pathways, actively repressing de novo synthesis [28]. Thus, when enterohepatic recycling is impaired—as observed in hyperuricemic models—the loss of both physical recovery and feedback inhibition leads to a compensatory increase in hepatic bile acid synthesis [13].

Evolutionary context of enterohepatic recycling. From an evolutionary standpoint, the efficient recycling of bile acids via enterohepatic circulation offers several adaptive advantages over continuous de novo synthesis. Bile acid synthesis from cholesterol is a multi-step, energy-intensive process; the rate-limiting step catalyzed by CYP7A1 alone consumes significant reducing equivalents (NADPH) and oxygen [36,37]. Recycling conserves these resources for other essential metabolic functions. In addition, continuous CYP7A1 expression would generate sustained oxidative stress due to cytochrome P450-mediated reactions, imposing cumulative cellular damage. The FXR-mediated feedback control—an ancient regulatory mechanism that acquired ligand specificity for bile acids late in vertebrate evolution [38,39]—allows rapid adjustments to changing dietary lipid loads without the transcriptional lag of de novo synthesis [40]. Notably, during pregnancy, maternal bile acid synthesis is physiologically downregulated while recycling efficiency is maintained or enhanced to support fetal and maternal metabolic needs [41]. This evolutionary design ensures that the limited bile acid pool (~2–4 g in humans) is reused approximately 20 times per day, enabling the body to maintain efficient lipid digestion with minimal biosynthetic expenditure.

Enterohepatic dysfunction in gout. A small amount of bile acids can enter the systemic circulation through non-classical routes and distribute to the kidneys, heart, adipose tissue, skeletal muscle and central nervous system, where they exert diverse endocrine and paracrine regulatory effects [42]. In the context of gout and hyperuricemia, total 12α-hydroxy bile acid levels are significantly elevated in hyperuricemic rat models [13]. This dysregulation may interfere with the normal FXR–FGF19 axis and, by altering gut microbiota composition, exacerbate intestinal barrier damage and systemic inflammation [16]. Therefore, targeting the efficiency of the enterohepatic circulation, reshaping the bile acid profile (especially the proportion of 12α-OH bile acids), or intervening in downstream signaling pathways has emerged as a novel therapeutic strategy for gout [14].

### 2.3. Bile Acid Signaling Receptors: FXR and TGR5

The biological effects of bile acids are primarily mediated through activation of specific receptors [43]. Among these, the farnesoid X receptor (FXR) and the G protein-coupled bile acid receptor 1 (TGR5) are the two best-characterized and functionally complementary cores, together constituting a dual axis that regulates metabolic and inflammatory homeostasis [7].

FXR: transcriptional regulation and anti-inflammation. FXR is a ligand-dependent transcription factor belonging to the nuclear receptor superfamily [44]. It is highly expressed in the liver, ileum and kidney, with functional expression also found in macrophages [45]. Conjugated or unconjugated primary bile acids (e.g., CDCA, CA) serve as the main ligands for FXR [44]. Upon activation, FXR suppresses CYP7A1 transcription by inducing SHP expression to negatively regulate bile acid synthesis [28], directly interferes with NF-κB transcriptional activity to inhibit the production of pro-inflammatory cytokines such as TNF-α, IL-1β and IL-6 [46], and upregulates negative regulators of the NLRP3 inflammasome (e.g., Sirtuin 2), thereby suppressing NLRP3 assembly and activation and blocking the maturation and release of IL-1β/IL-18 [47]. This mechanism is particularly relevant in MSU crystal-driven gouty inflammation [11].

TGR5: rapid signaling and metabolic integration. TGR5 is a membrane-associated G protein-coupled receptor with a broader tissue distribution, including intestinal L cells, brown adipose tissue, monocytes/macrophages, biliary epithelial cells and the central nervous system [6]. TGR5 has higher affinity for secondary bile acids (especially LCA and DCA) [48]. Its signaling proceeds via Gs protein activation, which elevates intracellular cAMP and activates the PKA pathway [10]. Upon bile acid binding, TGR5 couples to Gαs, activating adenylyl cyclase to convert ATP to cAMP. Elevated cAMP binds PKA regulatory subunits, releasing catalytic subunits for downstream phosphorylation. In intestinal L cells, this cAMP/PKA cascade activates CREB, driving proglucagon expression and GLP-1 secretion, with [Ca^2+^] elevation further potentiating release [14]. Activation of TGR5 promotes GLP-1 secretion from intestinal L cells to improve glucose homeostasis [49], stimulates thermogenesis in brown adipose tissue and energy expenditure in skeletal muscle [42], and suppresses NF-κB signaling while directly inhibiting NLRP3 inflammasome activity in macrophages, thereby reducing IL-1β production [10].

Expanded receptor network beyond FXR and TGR5. In addition to FXR and TGR5, other nuclear receptors have been shown to interact with specific bile acids [50]. The most hydrophobic secondary bile acid, LCA, is a potent ligand for the vitamin D receptor (VDR) and the pregnane X receptor (PXR), participating in bile acid detoxification and cytoprotection [51]. Moreover, certain bile acid metabolites can act as inverse agonists of the retinoid-related orphan receptor γt (RORγt), inhibiting Th17 cell differentiation and IL-17 production, thus playing a modulatory role in autoimmune and inflammatory diseases [52].

TGR5, VDR, PXR, and RORγt form an integrated receptor network: TGR5 provides rapid anti-inflammatory signals via cAMP/PKA; VDR and PXR maintain barrier integrity and detoxify harmful bile acids; RORγt controls the Th17/Treg balance. Certain microbial bile acids (e.g., 3-oxoDCA) act as dual TGR5 agonists and RORγt inverse agonists, illustrating receptor synergy [52]. In gout, reduced secondary bile acids impair this network, promoting barrier dysfunction and Th17-skewed inflammation [13]. Although these non-canonical pathways are less well understood than the FXR/TGR5 axis, they collectively depict a complex network through which bile acids finely regulate metabolism, immunity and barrier functions via multiple receptors and pathways [15]. In the context of gout, understanding the coordinated regulation of the NLRP3 inflammasome by these receptors provides a broad theoretical basis for developing novel anti-inflammatory and urate-lowering therapies targeting bile acid receptors [14]. A comprehensive summary of these components is provided in Table 1.

## 3. BA Modulates Uric Acid Production

### 3.1. PPAR-α Signaling Pathway: A Hub Linking Lipid Metabolism and Purine Metabolism

Peroxisome proliferator-activated receptor α (PPAR-α) is a ligand-activated nuclear receptor expressed predominantly in high-energy-demand tissues such as the liver, heart and skeletal muscle [53]. Traditionally viewed as a core transcription factor regulating fatty acid β-oxidation, recent studies have revealed its critical role in purine metabolism: PPAR-α has been shown to directly bind to the peroxisome proliferator response element (PPRE) in the promoter region of the xanthine oxidase (XOD) gene, thereby upregulating XOD transcription and enzymatic activity in preclinical studies [54]. XOD is the rate-limiting enzyme in the catabolism of purines to uric acid, catalyzing the sequential oxidation of hypoxanthine to xanthine and then to uric acid, and is the target of classic urate-lowering drugs such as allopurinol and febuxostat [2].

Under conditions of bile acid dysregulation, particularly when levels of hydrophobic primary bile acids (e.g., chenodeoxycholic acid, CDCA) or certain secondary bile acids (e.g., deoxycholic acid, DCA) are abnormally elevated, these bile acids are proposed to indirectly activate PPAR-α through FXR-independent pathways based on preclinical evidence (e.g., activation of the MAPK/ERK signaling pathway or induction of reactive oxygen species, ROS) [29,55]. This activation is suggested to lead to a marked increase in XOD expression in hepatocytes, thereby accelerating the conversion of purines to uric acid and potentially contributing to hepatic overproduction of uric acid—a proposed key pathogenic mechanism of hyperuricemia [3]. Thus, the PPAR-α/XOD axis is proposed as a potential hub linking disordered lipid metabolism and enhanced purine metabolism in the setting of bile acid dysregulation [4].

Molecular basis for PPAR-α activation by hydrophobic bile acids. Hydrophobic bile acids—particularly CDCA, DCA, and LCA—are especially implicated in PPAR-α modulation due to their physicochemical properties. Their greater lipophilicity, ordered as LCA > DCA > CDCA > CA, facilitates membrane partitioning and intracellular accumulation, enabling them to reach concentrations sufficient for receptor modulation. The molecular mechanisms linking bile acids to PPAR-α activation are complex and involve both FXR-dependent and FXR-independent pathways summarized in Table 2.

### 3.2. Indirect Effects of Bile Acid–Cholesterol Coupling on Uric Acid Levels

The tight coupling between bile acid and cholesterol metabolism exerts an indirect but important regulatory effect on uric acid levels, as convincingly demonstrated by studies on diosgenin [13]. Diosgenin, a natural steroidal sapogenin, acts as an inhibitor of the farnesoid X receptor (FXR) [57]. Upon activation by bile acids, FXR induces the expression of the small heterodimer partner (SHP), which in turn suppresses transcription of cholesterol 7α-hydroxylase (CYP7A1)—the rate-limiting enzyme in the conversion of cholesterol to bile acids [28]. By inhibiting FXR activation, diosgenin relieves SHP-mediated negative regulation of CYP7A1, thereby significantly upregulating CYP7A1 expression and activity and promoting the hepatic conversion of cholesterol into bile acids [14]. This process not only effectively reduces the body’s cholesterol pool but also, by alleviating excessive accumulation of hydrophobic bile acids, indirectly dampens overactivation of the PPAR-α/XOD pathway, and is associated with reduced serum uric acid levels in preclinical studies [13]. This “two birds with one stone” effect—simultaneously improving cholesterol dysmetabolism and hyperuricemia—provides a highly promising rationale for developing multi-target drugs for metabolic syndrome [7].

Furthermore, organic anion transporters (OATs), particularly OAT1 and OAT3 located on the basolateral membrane of renal proximal tubules, play a competitive role in the renal excretion of bile acids and uric acid, representing another important pathological link in hyperuricemia [58]. The primary function of OAT1/OAT3 is to take up various organic anions from the bloodstream into renal tubular epithelial cells for subsequent secretion into the urine [59]. Both uric acid and certain conjugated bile acids (e.g., glycochenodeoxycholate) are substrates of OAT1/OAT3 [60]. In states of cholestasis or hypercholanemia, circulating bile acid concentrations are markedly elevated, and these bile acids competitively bind to the transport sites of OAT1/OAT3, thereby inhibiting renal uric acid uptake and subsequent excretion [61]. This competitive inhibition leads to uric acid retention in the body, further exacerbating hyperuricemia caused by hepatic overproduction [62].

**Figure 1:**
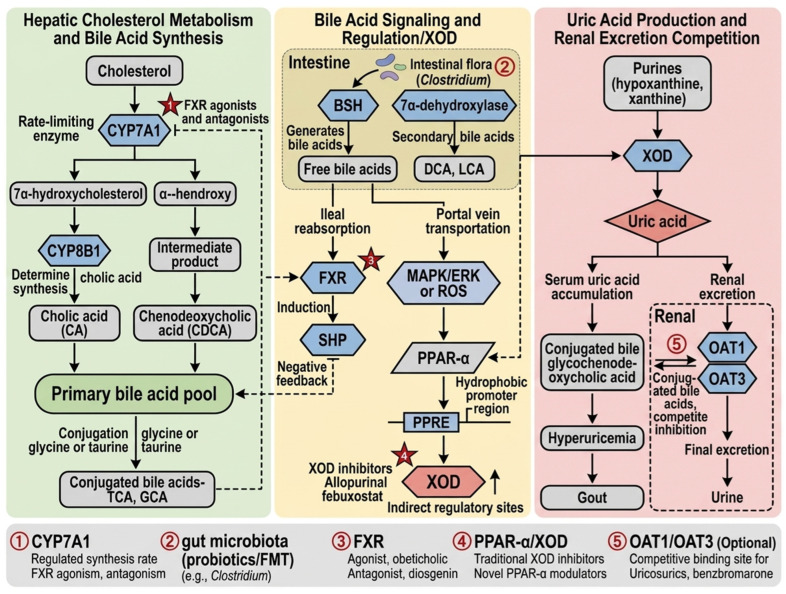
Integrated metabolic pathway from cholesterol to bile acid synthesis, PPAR-α/XOD-mediated uric acid production, and potential intervention targets. (**Left**) Hepatic cholesterol conversion to primary bile acids (CA, CDCA) via CYP7A1 and CYP8B1, followed by conjugation. (**Middle**) Gut microbiota (BSH, 7α-dehydroxylase) generate secondary bile acids (DCA, LCA), which signal via FXR (feedback inhibition of CYP7A1) or non-canonical pathways to activate PPAR-α, leading to upregulated XOD expression. (**Right**) XOD catalyzes uric acid production from purines; uric acid is excreted by renal OAT1/3, which also bind conjugated bile acids. Numbered red circles indicate potential therapeutic intervention points: ① CYP7A1 activity (via FXR); ② gut microbiota (probiotics/FMT); ③ FXR (agonists/antagonists); ④ PPAR-α/XOD axis; ⑤ OAT1/3 competition. Dashed lines represent inhibition; solid lines represent activation or conversion.

In summary, in the context of bile acid dysregulation, hyperuricemia often results from a “double hit”: on one hand, the liver may overproduces uric acid via the PPAR-α/XOD pathway (as suggested by preclinical findings); on the other hand, the kidney may reduce uric acid excretion due to competitive inhibition of OATs [1]. This complex pathophysiological network highlights the systemic and integrated nature of metabolic regulation and provides a theoretical foundation for understanding disease pathogenesis and developing comprehensive intervention strategies as shown in Figure 1 [15].

## 4. BA Suppresses NLRP3 Inflammasome via FXR/TGR5

### 4.1. MSU Crystal-Induced NLRP3 Inflammatory Cascade and Its Central Role in Gout

MSU crystals, as the direct trigger of gout flares, activate the NLRP3 inflammasome strictly according to a “two-signal” model [63]. First, pathogen- or damage-associated molecular patterns (PAMPs/DAMPs) in the joint microenvironment activate the Nuclear factor kappa-light-chain-enhancer of activated B cells (NF-κB) signaling pathway via Toll-like receptors, delivering “signal 1” [64]. This signal induces macrophages to transcribe and synthesize pro-IL-1β, pro-IL-18 and the NLRP3 protein itself, placing the cells in an “armed and ready” state [65]. Subsequently, MSU crystals provide “signal 2”: after phagocytosis of MSU crystals, the sharp crystals puncture the phagolysosomal membrane, leading to lysosomal content leakage, massive potassium efflux and a burst of reactive oxygen species (ROS) [66]. These events trigger NLRP3 oligomerization and assembly with ASC and pro-caspase-1 into a complete inflammasome complex [67]. Activated caspase-1 then cleaves pro-IL-1β and pro-IL-18 to generate the mature, highly pro-inflammatory cytokines IL-1β and IL-18, and induces gasdermin D-mediated pyroptosis, resulting in an intense sterile inflammatory response [68,69]. In this context, FXR activation has been shown to be a key negative regulatory node that suppresses this cascade: in macrophages, FXR agonists significantly inhibit MSU crystal-induced NLRP3 inflammasome assembly and subsequent IL-1β release [11].

### 4.2. FXR-Mediated Suppression of NLRP3

The inhibitory effect of FXR on the NLRP3 inflammasome is multi-layered and transcription-dependent [47]. First, FXR can directly bind to the promoter region of the NLRP3 gene and recruit co-repressor complexes to suppress its transcription, thereby reducing NLRP3 protein expression at the source [45]. Second, FXR physically interacts with the p65 subunit of the NF-κB signaling pathway, blocking p65 nuclear translocation and effectively inhibiting the expression of multiple inflammation-related genes (e.g., pro-IL-1β, TNF-α, IL-6) driven by signal 1 [46]. In addition, FXR activation promotes the production of anti-inflammatory cytokines such as IL-10, further shaping a microenvironment favorable to inflammation resolution [7]. Together, these mechanisms constitute a robust defense system in macrophages against MSU crystal-induced inflammation [15].

### 4.3. TGR5-Mediated Suppression of NLRP3

TGR5 is a G protein-coupled receptor expressed in intestinal L cells, macrophages and adipocytes [6]. Upon activation, it couples with Gαs protein to stimulate adenylyl cyclase, raising intracellular cAMP levels and subsequently activating protein kinase A (PKA) [48]. PKA can interfere with the proper assembly of the inflammasome complex by phosphorylating NLRP3 or ASC, thereby inhibiting its activity [10]. More importantly, TGR5 activation in intestinal L cells is a major pathway stimulating the secretion of GLP-1 [49]. GLP-1 is not only an important incretin that enhances glucose-stimulated insulin secretion and improves insulin resistance, but also possesses direct anti-inflammatory effects: GLP-1 activates the cAMP/PKA pathway via its receptor (GLP-1R), further suppressing NF-κB activation and NLRP3 inflammasome function in macrophages [70]. In patients with gout, the total bile acid pool and the levels of hydrophobic secondary bile acids (e.g., DCA, LCA, which are potent TGR5 agonists) are often reduced [13]. This bile acid dysregulation leads to insufficient TGR5 co-activation and consequently reduced GLP-1 secretion [14]. The decrease in GLP-1 levels weakens its suppressive effect on the NF-κB pathway and NLRP3 inflammasome, ultimately resulting in excessive release of pro-inflammatory cytokines such as IL-1β and TNF-α—forming a vicious cycle of “low bile acids → low TGR5 activity → low GLP-1 → high IL-1β → joint inflammation” [14].

Upon bile acid binding, TGR5 couples to Gαs, activating adenylyl cyclase to convert ATP to cAMP. Elevated cAMP binds PKA regulatory subunits, releasing catalytic subunits for downstream phosphorylation. In intestinal L cells, this cAMP/PKA cascade activates CREB, driving proglucagon expression and GLP-1 secretion, with [Ca^2+^] elevation further potentiating release [14].

### 4.4. Integrated Signaling Framework: Synergistic Role of the FXR–TGR5–NLRP3 Axis in Gout Immune Regulation

**Figure 2:**
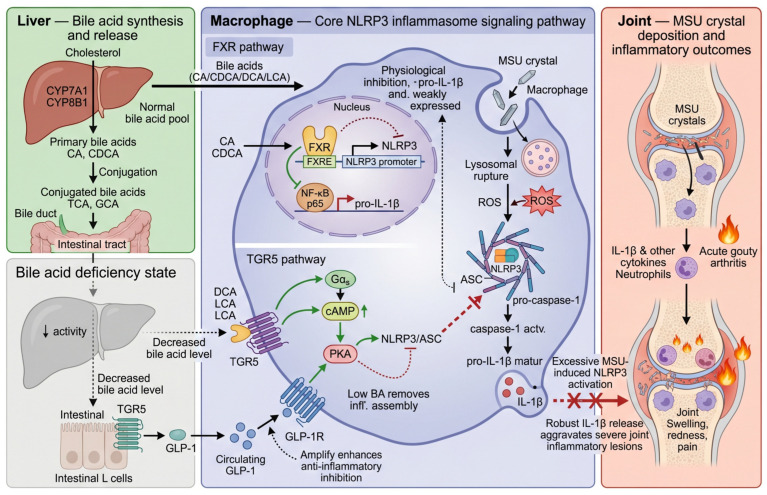
FXR/TGR5 suppress NLRP3 inflammasome in the liver–macrophage–joint axis during gout. Black solid arrows = induction/metabolic flux; red dotted arrows = transcriptional regulation; ↑ = increased levels; red bar symbols = pathway inhibition; red double crosses = severe downstream suppression; flame symbols = inflammatory injury.

FXR and TGR5 exhibit a refined division of labor and synergy in suppressing MSU crystal-induced inflammation [7]. FXR acts primarily at the transcriptional level as a “master planner”, limiting the supply of inflammatory “raw materials” by suppressing NLRP3 and pro-IL-1β gene expression [44]. In contrast, TGR5 provides “rapid intervention” at the signal transduction level, immediately blocking the assembly and activation of pre-existing proteins via the cAMP/PKA pathway and achieving systemic metabolic-immune regulation through GLP-1-mediated endocrine effects [42]. However, FXR function is markedly context-dependent, which is crucial for selecting therapeutic strategies [43]. In cholestasis or other high-bile-acid states, persistent and intense FXR activation in the liver induces the expression of the small heterodimer partner (SHP) [28]. SHP not only inhibits bile acid synthesis but also suppresses key signaling molecules such as insulin receptor substrate-2 (IRS-2), leading to hepatic insulin resistance and abnormal gluconeogenesis, and may promote the release of certain inflammatory factors—at which point FXR exhibits a pro-inflammatory tendency [71]. Conversely, in metabolic inflammatory conditions such as gout where bile acid levels are low, the body lacks sufficient endogenous FXR ligands, and the anti-inflammatory function of FXR cannot be effectively exerted [45]. Under these circumstances, administration of an appropriate FXR agonist can restore its ability to suppress the NF-κB/NLRP3 pathway and re-establish immune homeostasis, thereby conferring therapeutic benefit [11]. Thus, FXR-targeted therapy must be tailored to the metabolic context. In cholestasis, FXR antagonists or modulators may be appropriate, whereas in gout—a low-bile-acid state—dual FXR/TGR5 agonists or sequential regimens warrant investigation. One potential approach is to initiate TGR5 agonism to raise GLP-1 levels, followed by FXR activation to reinforce transcriptional suppression of inflammation as shown in Figure 2 [43]. The distinction between human-validated mechanisms and those supported by preclinical evidence is presented in Table 3.

## 5. Gut Microbiota–Bile Acid Bidirectional Crosstalk and Its Pathological Implications in Gout

### 5.1. Gut Microbiota-Mediated Chemical Modification of Bile Acids: A Key Determinant of the Secondary Bile Acid Profile

The gut microbiota exerts profound control over the composition and signaling functions of the bile acid pool through enzymatic modifications that are largely absent in the host genome. Two microbial enzymatic activities are of particular relevance to gout pathophysiology: bile salt hydrolase (BSH), which deconjugates taurine- or glycine-conjugated primary bile acids into free forms, and 7α-dehydroxylase, which converts free primary bile acids (CA and CDCA) into secondary bile acids (DCA and LCA)—the latter being potent TGR5 agonists (see Section 2.1 for detailed enzymatic chemistry). In patients with gout and hyperuricemia, the functional capacity of these microbial pathways is consistently diminished [13,25,26].

Metagenomic and metabolomic studies have revealed a characteristic signature of bile acid–modifying microbial dysfunction in gout: reduced abundance of BSH-active genera (particularly *Bifidobacterium* and *Lactobacillus*), depletion of 7α-dehydroxylase-producing *Clostridium* clusters (XIVa and XI), and a consequent decrease in fecal and serum DCA and LCA levels [13,72,73]. This functional deficit is not merely correlative—it directly impairs TGR5-mediated anti-inflammatory signaling and FXR-dependent metabolic regulation, establishing a mechanistic link between microbial ecology and gout pathogenesis [7,14]. Importantly, the reduction in secondary bile acids also weakens the antimicrobial barrier function of the bile acid pool, creating permissive conditions for opportunistic pathogens to expand and further destabilize the ecosystem.

### 5.2. Bile Acid-Mediated Regulation of the Gut Microbiota: Antimicrobial Selection Pressure and Ecological Balance

Bile acids are not passive substrates for microbial modification; they actively shape gut microbial community structure through direct antimicrobial activity [74]. At physiological concentrations, hydrophobic secondary bile acids (DCA and LCA) disrupt bacterial membrane integrity and suppress the overgrowth of Gram-positive organisms and certain Gram-negative bacteria [75]. This selective pressure maintains a balanced microbial ecosystem in which beneficial commensals (e.g., butyrate producers) coexist with bile-acid-tolerant taxa [76].

In gout, however, the contracted bile acid pool and reduced DCA/LCA levels weaken this antimicrobial selection pressure [13]. The resulting ecological release allows expansion of opportunistic pathogens—notably *Fusobacterium*, *Bilophila*, and certain *Enterobacteriaceae*—that are ordinarily held in check by hydrophobic bile acids [12,72]. Concurrently, the abundance of short-chain fatty acid (SCFA)-producing commensals—*Faecalibacterium prausnitzii*, *Coprococcus*, *Anaerostipes*, and *Lachnospira*—declines significantly [16,72]. This shift has functional consequences: SCFAs (particularly butyrate) not only provide energy for colonic epithelial cells and maintain intestinal barrier integrity but also suppress NLRP3 inflammasome activation through inhibition of class I histone deacetylases [77,78]. Thus, the loss of SCFA-producing taxa amplifies the inflammatory vulnerability of gout patients through a microbial pathway independent of bile acids themselves [16].

### 5.3. Specific Alterations in the Context of Hyperuricemia/Gout: The 12α-OH Bile Acid Profile and Microbiota Associations

**Figure 3:**
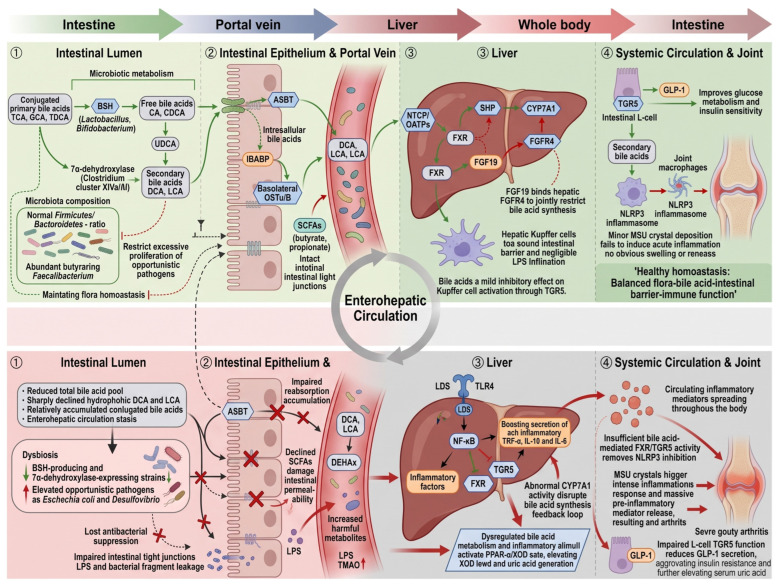
Gut–portal vein–liver–systemic circulation axis: bidirectional bile acid–microbiota crosstalk in gout pathogenesis and rationale for microbiota-targeted interventions. (**Upper**) Normal bile acid pool and composition support a balanced microbiota via antimicrobial effects (DCA/LCA). Bile acids are efficiently reabsorbed via ASBT, and gut barrier integrity is maintained by SCFAs. Hepatic FXR/FGF19 feedback regulates bile acid synthesis, and Kupffer cells remain quiescent. Systemic bile acids activate TGR5 in L cells (GLP-1) and joint macrophages (NLRP3 suppression). (**Lower**) Reduced total bile acids and decreased DCA/LCA lead to dysbiosis. Impaired antimicrobial activity and reduced SCFAs compromise gut barrier integrity, allowing LPS translocation into the portal vein. In the liver, LPS activates Kupffer cells, producing inflammatory cytokines that further suppress FXR/TGR5 expression. Hepatic PPAR-α/XOD upregulation increases uric acid production. Green solid arrows = normal metabolic transport and positive regulation; black dotted arrows = indirect regulatory effects; red crosses = disrupted physiological transport/pathway blockade; ↑ = increased molecular abundance; circular arrow = enterohepatic recirculation.

The bidirectional interactions described above converge on a self-reinforcing pathological cycle that is central to gout progression [7,14]. The cycle initiates with hyperuricemia-associated dysbiosis—characterized by depletion of BSH- and 7α-dehydroxylase-active bacteria and expansion of pro-inflammatory opportunists [13,72]. Reduced microbial modification of bile acids leads to a contracted pool of TGR5-active secondary bile acids (DCA/LCA), weakening FXR/TGR5 signaling and diminishing the antimicrobial barrier function of the bile acid pool [48,74]. The loss of antimicrobial selection pressure permits further dysbiosis, while compromised SCFA production impairs intestinal epithelial barrier integrity—increasing permeability to bacterial endotoxin (lipopolysaccharide, LPS) [79]. LPS translocation into the portal circulation primes hepatic Kupffer cells and systemic macrophages via TLR4/NF-κB activation, providing “signal 1” for NLRP3 inflammasome licensing [63]. This pro-inflammatory state further suppresses bile acid synthesis and transport, closing the cycle and perpetuating both metabolic and inflammatory pathology as shown in Figure 3 [7,14].

What distinguishes this cycle in gout from other metabolic diseases is the specific confluence of: (a) the 12α-hydroxylated bile acid predominance—CA and its conjugates accumulate while DCA/LCA decline, reflecting both hepatic overproduction and microbial underconversion [13]; (b) the depletion of *Clostridium* clusters XIVa and XI—the principal 7α-dehydroxylase carriers in the human gut [25]; and (c) the expansion of LPS-bearing Gram-negative opportunists (e.g., *Bilophila*, *Fusobacterium*) that directly fuel the inflammasome-priming step [12,72]. These features collectively position the microbiota–bile acid–inflammation cycle as a gout-specific amplification loop that distinguishes it from the microbial alterations observed in obesity or type 2 diabetes alone.

### 5.4. Gut Microbial Alterations in Gout: A Functionally Annotated Profile

To provide a structured overview of consistently reported gut microbial changes in gout, we summarize the key taxa, their functional roles, and bile acid–related implications in Table 4. The table is organized by functional category—SCFA producers, BSH-active taxa, and pro-inflammatory opportunists—rather than alphabetical listing, to highlight the functional consequences of dysbiosis.

## 6. Bile Acid Profile in Gout and Hyperuricemia

### 6.1. Bile Acid Profile Abnormalities in Clinical Studies: Total Amount, Composition, and Transformation Characteristics

Patients with gout and hyperuricemia exhibit complex and characteristic alterations in their bile acid profile [4]. These changes not only reflect a profound disturbance of the metabolic network in disease states but also provide important clues for understanding pathogenesis and developing novel intervention strategies [1]. Integrating data from clinical cohort studies and animal models, abnormalities in the bile acid profile are mainly manifested in total amount, compositional ratios, and enterohepatic circulation efficiency [13]. In clinical studies, serum or plasma metabolomic analyses of gout patients have revealed significant disturbances in bile acid metabolism [9]. Although the direction of change in total bile acid (TBA) levels varies across studies, most evidence tends to support a decreasing trend, which may result from impaired hepatic synthesis capacity, intestinal reabsorption defects, or systemic inflammation-induced suppression of bile acid transporters [30]. A more consistent finding is a marked perturbation of the primary bile acid biosynthesis pathway: levels of the primary bile acid cholic acid (CA) are often reported to be elevated, whereas levels of the secondary bile acid deoxycholic acid (DCA), generated by the gut microbiota via 7α-dehydroxylation, are generally decreased [8]. The reduction in DCA directly reflects gut dysbiosis, particularly a decline in the abundance of DCA-producing *Clostridium* species [25]. Concurrently, the proportion of conjugated bile acids (e.g., taurocholic acid, TCA; glycocholic acid, GCA) is relatively increased, possibly due to altered hepatic BAAT activity or impaired intestinal deconjugation [21]. These compositional changes reduce the hydrophobicity and signaling potency of the bile acid pool, thereby affecting the activation status of FXR and TGR5 [7]. Notably, ursodeoxycholic acid (UDCA), a hydrophilic bile acid with cytoprotective and anti-inflammatory properties, has shown preliminary efficacy in hyperuricemic patients with comorbid non-alcoholic fatty liver disease (NAFLD) [80]. UDCA not only improves liver function tests and hepatic steatosis but also concomitantly lowers serum uric acid levels, possibly through inhibition of XOD activity, attenuation of hepatocellular inflammation, and modulation of the bile acid–FXR axis to restore metabolic homeostasis [13].

Although most studies support the conclusion of a reduced total bile acid pool and decreased secondary bile acids (DCA, LCA) in gout patients, several investigations have reported inconsistent or divergent findings. For instance, while Zhong et al. identified primary bile acid biosynthesis as a significantly altered pathway in gout patients, their study did not report a uniform direction of change across all bile acid species [83]. Similarly, Jia et al. found that the urinary metabolic signatures of acute and chronic gout patients were primarily involved in purine metabolism and amino acid metabolism, with less pronounced alterations in bile acid pathways compared to serum-based studies, suggesting that the choice of biofluid (serum vs. urine) may substantially influence the detected bile acid profile [8]. Huang et al. further demonstrated that serum metabolomics provided a clearer distinction between gout patients and healthy controls than urine metabolomics, and identified bile secretion as one of the involved pathways—yet the specific bile acid species and their direction of change were not uniformly consistent across cohorts [86]. These discrepancies may arise from differences in patient baseline characteristics (e.g., dietary habits, urate-lowering medication use, comorbidities such as obesity and NAFLD), sample types (serum, urine, or feces), detection platforms (targeted vs. untargeted metabolomics), and disease phases (acute flare vs. intercritical period). Furthermore, the causal relationship between 12α-hydroxy bile acids and uric acid levels currently rests primarily on animal model evidence, and large-scale prospective cohort studies in humans are lacking to validate these findings [14,85]. Therefore, interpretation of current bile acid profile alterations in gout should be approached with caution, with due consideration of the substantial heterogeneity across studies.

### 6.2. Animal Model Studies: Hyperuricemia Actively Drives Bile Acid Metabolic Alterations

Animal model studies have further elucidated how hyperuricemia actively drives alterations in bile acid metabolism [14]. In rat models of hyperuricemia, elevated hepatic CA levels clearly indicate upregulation of de novo bile acid synthesis, which may represent a compensatory response to metabolic stress [19]. However, this compensation does not effectively restore bile acid function [3]. In contrast, marked accumulation of conjugated bile acids (e.g., TCA, GCA) in the ileal content strongly suggests a “stagnation” of the enterohepatic circulation of bile acids [35]. Under normal conditions, approximately 95% of bile acids are efficiently reabsorbed in the terminal ileum and returned to the liver via the portal vein; the accumulation of conjugated bile acids in the ileal content indicates impaired reabsorption, possibly due to downregulation or dysfunction of the apical sodium-dependent bile acid transporter (ASBT) in the ileal brush border [34]. Furthermore, elevated levels of total 12α-hydroxy bile acids have been observed [13]. Since 12α-hydroxylation is a critical step in CA synthesis, and the activity of this pathway is potentially linked to XOD-mediated uric acid production, 12α-OH bile acids are considered important biomarkers connecting purine metabolism and bile acid metabolism [54]. These findings indicate that hyperuricemia is not an isolated metabolic abnormality but can profoundly disrupt the efficiency of enterohepatic circulation and the signaling functions of bile acids, primarily through the 12α-OH bile acid pathway summarized in Table 5 [15].

### 6.3. Human Clinical Evidence on Bile Acid Metabolism in Gout Patients

Beyond the descriptive profiling of bile acid alterations, several human clinical studies have begun to explore the functional relevance of bile acid metabolism in gout pathophysiology. Zhong et al. [83] conducted a serum metabolomic analysis of 31 gout patients and 31 healthy controls using UPLC-Q-TOF/MS, and identified primary bile acid biosynthesis as a significantly altered pathway in gout, alongside purine and glycerophospholipid metabolism. Huang et al. [86] further demonstrated, in a discovery cohort of 30 gout patients and 30 controls with an independent validation cohort of 100 participants, that serum metabolomics provided a clearer distinction than urine metabolomics; pathway analysis implicated bile secretion, purine metabolism, branched-chain amino acid metabolism, and arachidonic acid metabolism in gout pathogenesis. Importantly, Wang et al. [87] performed a large-scale metabolomics study comparing 239 patients with frequent gout flares versus 163 patients with infrequent flares, and identified bile acids among the top dysregulated pathways—alongside carbohydrates, amino acids, and nucleotide metabolism—that distinguished flare frequency. This finding suggests that bile acid metabolic disturbances may not only differentiate gout patients from healthy individuals but also correlate with disease activity and clinical outcomes.

In addition, the Xiangya Hyperuricemia Study [88], a community-based investigation employing shotgun metagenomic sequencing, examined the relationship between gut microbiome, bile acid compositions, and prevalent hyperuricemia, providing evidence that the gut microbiota–bile acid axis may mediate the development of hyperuricemia. A prospective study [84] further demonstrated that dietary lignan intake was inversely associated with hyperuricemia incidence in middle-aged and elderly Chinese individuals, and this association was potentially mediated by the gut microbiota–bile acid axis.

Collectively, these human studies consistently implicate bile acid metabolism—particularly primary bile acid biosynthesis and bile secretion pathways—in gout pathogenesis and flare susceptibility. However, it must be emphasized that the vast majority of these findings derive from cross-sectional metabolomic analyses, which are inherently limited in establishing causal relationships. Whether the observed bile acid alterations are drivers of gout pathogenesis or merely secondary epiphenomena of systemic metabolic disturbances and inflammation remains to be determined. Prospective cohort studies with pre-diagnostic metabolomic profiling and longitudinal follow-up are urgently needed to clarify the temporal sequence and causal direction of these associations.

## 7. Therapeutic Strategies Targeting the Bile Acid Pathway: A Multi-Target, Multi-Level New Frontier in Gout Management

### 7.1. FXR-Targeted Strategies: Dual Potential and Intrinsic Complexity

As the master regulator of bile acid metabolism, FXR presents both therapeutic potential and intrinsic pharmacological complexity [15]. In the context of gout, whether FXR should be activated or inhibited depends on the specific pathophysiological state [43]. The effects of FXR activation are highly tissue-dependent, and this tissue specificity is central to understanding its therapeutic potential and limitations in gout. In the liver, FXR activation induces SHP, which suppresses CYP7A1 and reduces the bile acid pool—an effect that may be undesirable in gout, where bile acid levels are already low. In macrophages, however, FXR activation provides a critical anti-inflammatory brake by suppressing NLRP3 transcription via direct promoter binding and NF-κB p65 interference. In the intestine, FXR induces FGF19, which signals back to the liver to regulate metabolic homeostasis. Thus, an ideal FXR-directed therapy for gout would need to engage the anti-inflammatory pathway in immune cells while sparing or minimizing the hepatic CYP7A1-suppressive effects—a challenge that selective FXR modulators and tissue-restricted agonists seek to address.

#### 7.1.1. FXR Agonists: Anti-Inflammatory Benefits vs. Metabolic Costs

FXR agonists (e.g., obeticholic acid, GW4064) lower blood lipids, improve non-alcoholic fatty liver disease (NAFLD), and suppress NLRP3 inflammasome transcription and activation in macrophages [11]. Mechanistically, FXR binds to FXREs in the NLRP3 promoter, interacts with the NF-κB p65 subunit, and upregulates negative regulators such as Sirtuin 2, thereby reducing pro-IL-1β and NLRP3 protein expression at the source [47]. For gout patients with concomitant metabolic syndrome (obesity, insulin resistance, NAFLD), FXR agonists may offer dual benefits [4].

However, this approach carries inherent trade-offs. FXR activation strongly induces small heterodimer partner (SHP) expression, which directly suppresses CYP7A1 transcriptional activity, leading to reduced de novo bile acid synthesis [28]. This further lowers circulating levels of hydrophobic secondary bile acids (e.g., DCA, LCA), which are potent endogenous TGR5 ligands [48]. Thus, long-term FXR agonism may attenuate TGR5-mediated GLP-1 secretion and anti-inflammatory effects—an unfavorable compromise [49]. Moreover, excessive FXR activation can suppress insulin receptor substrate-2 (IRS-2) via SHP, exacerbating hepatic insulin resistance [71]. These trade-offs argue against the use of pure FXR agonists as monotherapy in gout [14].

#### 7.1.2. FXR Antagonists: Promoting Bile Acid Synthesis but Potentially Compromising Anti-Inflammation

FXR antagonists (e.g., diosgenin, guggulsterone) relieve SHP-mediated inhibition of CYP7A1, thereby promoting conversion of cholesterol to bile acids, increasing the total bile acid pool, and raising the proportion of hydrophobic secondary bile acids—potentially enhancing TGR5 signaling indirectly [57]. On the other hand, antagonizing FXR weakens its direct anti-inflammatory functions, including transcriptional suppression of NF-κB and NLRP3, which may exacerbate MSU crystal-induced inflammation [46]. Therefore, a pure FXR antagonist is unsuitable for gout unless it possesses additional anti-inflammatory mechanisms [3].

#### 7.1.3. Alternative Approaches: FXR Modulators and Dual-Function Molecules

Recent work on benzbromarone derivatives illustrates a new therapeutic approach [14]. Benzbromarone itself is a URAT1 inhibitor used to promote uric acid excretion [82]. Researchers have optimized its structure to obtain novel molecules that both inhibit URAT1 (lowering serum urate) and activate FXR (exerting anti-inflammatory effects) [12]. This dual-mechanism approach avoids the limitations of pure agonism or antagonism—moderate FXR activation is sufficient for anti-inflammation without excessive CYP7A1 suppression [7]. Additionally, selective FXR modulators (partial agonists or biased ligands) that activate only anti-inflammatory downstream pathways without disturbing bile acid synthesis warrant further investigation [45].

### 7.2. TGR5 Agonists: Anti-Inflammatory Potential and Need for Clinical Validation

Compared with FXR, TGR5—being a membrane receptor that does not directly regulate bile acid synthesis—provides anti-inflammatory effects without directly regulating bile acid synthesis [42].

#### 7.2.1. Direct Inhibition of NLRP3 Inflammasome Assembly

Activation of TGR5 in macrophages and monocytes, via the Gαs-cAMP-PKA signaling pathway, directly phosphorylates NLRP3 or ASC, interfering with MSU crystal-induced inflammasome complex assembly and effectively reducing the release of key pro-inflammatory cytokines such as IL-1β and IL-18 [10]. TGR5 agonists (e.g., INT-777, oleanolic acid) reduce IL-1β in supernatants of MSU-stimulated macrophages; this effect is blocked by TGR5 siRNA or PKA inhibitors [10].

#### 7.2.2. Systemic Anti-Inflammation Through GLP-1

Activation of TGR5 in intestinal L cells promotes the secretion of glucagon-like peptide-1 (GLP-1) [48]. GLP-1 is not only an incretin that improves insulin resistance and glucose metabolism but also acts via the GLP-1 receptor (GLP-1R) on macrophages to activate the cAMP/PKA pathway, further suppressing NF-κB activation and NLRP3 inflammasome function [70]. Thus, TGR5 agonists exert anti-inflammatory effects through both direct action on immune cells and GLP-1-mediated systemic signaling [13].

#### 7.2.3. Outstanding Issues

Although TGR5 agonists have demonstrated efficacy in various metabolic inflammation models (e.g., fatty liver, atherosclerosis, colitis), their efficacy and safety in gout-specific animal models (e.g., MSU crystal-induced ankle gouty arthritis) and in clinical gout patient cohorts remain unconfirmed [8]. Several questions require investigation: whether TGR5 agonists relieve acute gout symptoms, whether long-term use causes tachycardia (a known side effect of some TGR5 agonists), and whether they are suitable for combination with existing urate-lowering drugs (allopurinol, febuxostat, benzbromarone). These questions must be addressed before clinical application [1].

### 7.3. Microecological Agents: Non-Pharmacological Interventions to Reshape the Microbiota–Bile Acid–Inflammation Axis

Microecological agents, including probiotics, prebiotics, and fecal microbiota transplantation (FMT), offer non-pharmacological means to modulate the microbiota–bile acid–inflammation axis [72]. Their advantage lies in restoring endogenous bile acid signaling rather than providing exogenous ligands [25].

#### 7.3.1. Probiotics: Modulating the Bile Acid Profile and Intestinal Barrier

Specific probiotic strains (e.g., *Lactobacillus*, *Bifidobacterium*) express bile salt hydrolase (BSH), which hydrolyzes conjugated bile acids (TCA, GCA, TDCA, etc.) into free bile acids (CA, CDCA, etc.), altering the composition of the bile acid profile and increasing the proportion of free bile acids [81]. Free bile acids (especially CA and CDCA) are effective FXR ligands, and their further 7α-dehydroxylation by the microbiota generates DCA and LCA, which are potent TGR5 agonists [74]. Thus, supplementation with BSH-producing probiotics can indirectly enhance both FXR and TGR5 signaling [89].

In addition, these probiotics restore intestinal epithelial barrier function compromised by hyperuricemia or antibiotics: they competitively inhibit pathogen adhesion, promote expression of tight junction proteins (e.g., occludin, claudin), and increase mucus secretion, thereby reducing lipopolysaccharide (LPS) translocation and blunting NF-κB priming for NLRP3 inflammasome activation [79]. Recent rat model studies indicate that probiotic intervention reshapes gut microbiota structure (e.g., increasing butyrate-producing bacteria, reducing Proteobacteria abundance) and simultaneously improves purine metabolism (lowering xanthine oxidase activity), tryptophan metabolism (increasing AhR ligands), and the bile acid profile (raising DCA/LCA levels), achieving multi-dimensional restoration of metabolic homeostasis [77].

#### 7.3.2. Prebiotics and Synbiotics

Prebiotics (e.g., fructooligosaccharides, inulin, resistant starch) selectively promote the growth of BSH- and butyrate-producing bacteria, indirectly enhancing bile acid deconjugation and secondary bile acid generation [78]. Synbiotics (probiotics + prebiotics) have shown superior effects to single agents in hyperuricemia animal models [90].

#### 7.3.3. Fecal Microbiota Transplantation (FMT)

FMT has a strong mechanistic rationale—transplanting a complete healthy donor microbiota into a patient could theoretically restore normal bile acid metabolism and immune regulation in one intervention [76]. However, FMT faces substantial challenges before clinical adoption, including standardization of donor screening (excluding potential pathogens and metabolic disease risks), long-term safety assessment (e.g., unexpected metabolic alterations), durability of efficacy (single FMT has limited duration), and ethical/regulatory issues [75]. No clinical trial of FMT in gout patients has been reported to date, and its clinical utility remains unestablished [91].

### 7.4. Traditional Chinese Medicine and Natural Products as Bile Acid Modulators

Traditional Chinese medicine (TCM) and natural products are a resource for discovering novel bile acid modulators [80]. Many studies have shown that certain TCM formulas or monomeric ingredients can enhance bile acid synthesis by improving gut microbiota structure and activating CYP7A1, thereby ameliorating gout-related inflammation [13].

#### 7.4.1. Diosgenin

Diosgenin is a natural steroidal sapogenin found abundantly in plants such as yam (*Dioscorea* species) [13]. Studies have shown that diosgenin, as an FXR antagonist, inhibits excessive FXR activation and relieves the negative regulation of CYP7A1, thereby promoting hepatic conversion of cholesterol into bile acids [57]. This process not only lowers serum cholesterol levels but also, by increasing the total bile acid pool and modulating bile acid composition, indirectly suppresses the PPAR-α/XOD pathway (because certain bile acids can act as natural PPAR-α ligands or affect its activity through FXR-independent mechanisms), ultimately leading to a significant reduction in serum uric acid levels [29]. This dual urate-lowering and anti-inflammatory profile provides a lead structure for developing novel therapeutics [13].

#### 7.4.2. Other Natural Products

Berberine modulates gut microbiota, increases the abundance of BSH-active bacteria, and directly activates TGR5, displaying urate-lowering and anti-inflammatory effects in various metabolic inflammation models [9]. Resveratrol improves bile acid metabolism and inhibits NLRP3 inflammasome via activation of the SIRT1–PPAR-α axis [53]. Curcumin modulates the FXR/TGR5 balance and directly inhibits NF-κB and NLRP3 activation [68].

#### 7.4.3. Challenges and Prospects

Natural product research is limited by low bioavailability (most compounds are poorly water-soluble and easily metabolized), undefined effective doses, and insufficient high-quality clinical trials [60]. Moreover, the multi-component, multi-target nature of TCM formulas complicates mechanistic analysis [61]. Future studies may employ systems pharmacology and metabolomics to investigate the causal relationships between active ingredient clusters, targets, and bile acid profiles [62].

### 7.5. Bile Acid Replacement Therapy: Ursodeoxycholic Acid

Bile acid replacement therapy, particularly with ursodeoxycholic acid (UDCA), is discussed here [17]. UDCA is a hydrophilic, non-toxic secondary bile acid traditionally used for primary biliary cholangitis (PBC) and gallstones [18]. Preliminary applications in hyperuricemic patients with comorbid NAFLD have shown improvements in liver function (reduced ALT, AST) and a concomitant reduction in serum uric acid levels [80]. Potential mechanisms include:

(a) direct or indirect inhibition of XOD activity, reducing uric acid production [2];
(b) stabilization of mitochondrial membranes and suppression of endoplasmic reticulum stress, downregulating inflammatory cytokines such as TNF-α and IL-6 and improving the liver microenvironment [21];
(c) remodeling of the bile acid pool composition (increasing hydrophilic/decreasing hydrophobic ratio), indirectly modulating FXR and TGR5 activity and restoring metabolic homeostasis [24].

Nevertheless, the mechanisms remain incompletely defined [35]. Key unresolved questions include whether UDCA directly engages FXR/TGR5–NLRP3 signaling, whether its effects are direct or secondary to bile acid pool remodeling, whether it is effective in hyperuricemic patients without NAFLD, and what the optimal dose and duration are. These questions will define the role of UDCA in gout management [34].

### 7.6. Clinical Translation Landscape of Bile Acid–Targeted Therapies

**Figure 4:**
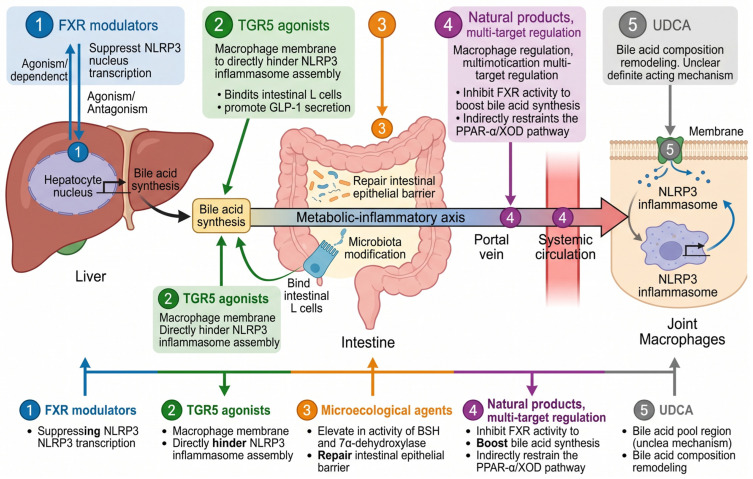
Target–intervention–outcome framework for bile acid-related strategies in gout.

To bridge the gap between preclinical discoveries and clinical application, it is essential to survey the current status of clinical trials, safety profiles, dosing considerations, and regulatory pathways for bile acid–targeted interventions as shown in Figure 4 [15]. Table 6 summarizes the key therapeutic agents, their development phases, target indications, and safety considerations.

#### 7.6.1. FXR Agonists

Obeticholic acid (OCA, INT-747), a semi-synthetic derivative of chenodeoxycholic acid with approximately 100-fold greater potency than the natural ligand, is the first-in-class FXR agonist to receive regulatory approval [92]. In 2016, OCA gained FDA approval for the treatment of primary biliary cholangitis (PBC) as a second-line therapy for patients with inadequate response or intolerance to ursodeoxycholic acid. The approved starting dose is 5 mg once daily, titrated to 10 mg based on tolerability, with dose adjustments required in patients with moderate to severe hepatic impairment. However, OCA has not been approved for any metabolic indication; phase 3 trials in non-alcoholic steatohepatitis (NASH) (e.g., REGENERATE trial, NCT03439254) have yielded mixed results, and regulatory decisions remain pending [93].

The safety profile of FXR agonists warrants careful consideration. A meta-analysis of FXR agonist trials in metabolic dysfunction-associated steatotic liver disease (MASLD) found that FXR agonists were associated with a significantly higher incidence of pruritus (37.8% vs. 18.7% in controls; RR 2.67, 95% CI 1.63–4.38, *p* < 0.00001), leading to higher treatment discontinuation rates [94]. A real-world pharmacovigilance study of OCA identified pruritus (12.54%), fatigue (4.16%), and nausea (1.64%) as the most prevalent adverse events [95]. Importantly, 17% of patients discontinued OCA treatment, with pruritus accounting for 36.9% of discontinuations [96]. Additional safety concerns include dose-dependent increases in LDL cholesterol and reductions in HDL cholesterol, as well as potential hepatic complications, particularly in patients with pre-existing cirrhosis. These safety issues have prompted the development of second-generation FXR agonists with improved therapeutic windows, including MET409 (phase 1b, showing lower risk of pruritus and LDL-c increment compared to OCA), cilofexor (phase 2, NCT02854605), tropifexor (phase 2, NCT03517540), and the non-bile acid FXR agonist HPG1860 (phase 2a, NCT05338034). INT-787, a novel hydrophilic semisynthetic bile acid FXR agonist, has recently completed a first-in-human phase 1 trial evaluating safety, tolerability, and pharmacokinetics in healthy volunteers.

Importantly, no FXR agonist has been approved for NAFLD/NASH to date, and none has been specifically evaluated in gout or hyperuricemia populations in clinical trials. Whether the anti-inflammatory benefits observed in preclinical MSU-induced arthritis models will translate to gout patients remains an open question requiring dedicated clinical investigation.

#### 7.6.2. TGR5 Agonists

In contrast to FXR, the clinical development of TGR5 agonists has been more limited. Several synthetic and semisynthetic TGR5 agonists have been advanced into early-phase clinical trials, primarily for type 2 diabetes mellitus (T2DM), obesity, and NAFLD [97]. SB756050, a selective TGR5 agonist developed by GlaxoSmithKline, entered phase 1 trials for T2DM but was ultimately discontinued after phase 2 assessments due to failure to demonstrate the expected pharmacodynamic profile. BAR502, a dual FXR/TGR5 agonist, has been advanced into early-phase clinical trials due to its favorable pharmacokinetic properties. Currently, only a few molecules targeting TGR5 remain in phase 1 and phase 2 clinical trials, primarily for metabolic and inflammatory indications.

A major obstacle in TGR5 agonist development is the risk of on-target side effects resulting from systemic receptor activation. TGR5 is highly expressed in the gallbladder and cholangiocytes, and systemic TGR5 activation inhibits gallbladder emptying, leading to gallbladder filling, bile stasis, and increased risk of gallstone formation. Additionally, TGR5 activation in cardiovascular tissues has been associated with reduced blood pressure and reflex tachycardia. These safety concerns have become the main barrier to the clinical advancement of systemically available TGR5 agonists. Emerging strategies to circumvent these issues include the development of gut-restricted TGR5 agonists that activate the receptor in intestinal L cells (to stimulate GLP-1 secretion) without systemic exposure, and “soft drugs” designed for rapid clearance from the circulation. Notably, no TGR5 agonist has been evaluated in gout-specific clinical trials to date.

#### 7.6.3. Microbiota-Based Interventions

Several clinical trials have investigated probiotic supplementation in hyperuricemia and gout. A two-month randomized, double-blind, placebo-controlled trial evaluated the additional benefit of co-administering Probio-X (a multi-strain probiotic formulation) alongside febuxostat in gout patients, with baseline gut microbiome composition being investigated as a predictive biomarker of response [98]. While this trial does not directly measure bile acid endpoints, it represents an important step toward evidence-based microbiota modulation in gout management.

#### 7.6.4. UDCA and Related Bile Acid Therapies

Ursodeoxycholic acid (UDCA) has a well-established safety profile, having been approved for PBC and gallstone dissolution for decades. The standard dose ranges from 10–15 mg/kg/day, and the drug is generally well-tolerated with minimal adverse effects. A prospective study in 53 NAFLD patients demonstrated that 6 months of UDCA therapy (12 mg/kg/day) significantly reduced serum uric acid from 386 to 334 μmol/L (*p* = 0.001), alongside improvements in liver fat fraction, total cholesterol, and triglycerides [99]. No significant side effects were observed. However, UDCA has not been evaluated in randomized controlled trials specifically designed for gout or hyperuricemia without coexisting NAFLD. The urate-lowering effect observed in NAFLD patients may reflect improvement in underlying metabolic dysfunction rather than a direct pharmacological action on uric acid metabolism, and this distinction requires further investigation.

#### 7.6.5. Regulatory and Translational Considerations

Several key observations emerge from this clinical translation landscape. First, while FXR is a validated drug target (OCA is FDA-approved for PBC), the repurposing of FXR agonists for gout faces substantial hurdles, including the lack of gout-specific clinical trial data, the context-dependent nature of FXR signaling (where activation may be beneficial in low-bile-acid states but problematic in cholestasis), and the significant safety concerns (pruritus, dyslipidemia) that may limit long-term use in relatively healthy gout populations. Second, TGR5 agonists remain at an early stage of clinical development, with no agents having progressed beyond phase 2 for any indication, and systemic safety concerns (gallbladder and cardiovascular effects) posing major obstacles. Third, the most clinically advanced bile acid–related intervention for urate lowering is UDCA, but its efficacy in gout patients without NAFLD and its mechanism of action require definitive validation. Fourth, the development of next-generation agents—including gut-restricted TGR5 agonists, selective FXR modulators with improved safety profiles, and dual-target molecules (e.g., URAT1/FXR dual inhibitors)—represents the most promising pathway toward clinical translation. Dedicated phase 2/3 trials in gout patients, with appropriate patient stratification based on bile acid profiles and comorbidities, will be essential to determine whether the promising preclinical findings summarized in this review can be successfully translated into clinical practice. The clinical development status, safety profiles, and dosing considerations for these agents are summarized in Table 7.

## 8. Summary and Future Perspectives

### 8.1. Integrated Framework: From Metabolic Disruption to Inflammatory Exacerbation

The preceding sections establish a paradigm-shifting framework in which bile acids transcend their canonical role in lipid emulsification to function as central integrators of purine metabolism and immune surveillance within the gut–liver–joint axis [5,6,100]. In essence, the pathogenesis of gout under this lens converges on a self-reinforcing triangular pathology: hepatic overproduction of urate driven by PPAR-α/XOD upregulation [29,55], NLRP3 inflammasome hyperactivation disinhibited by diminished FXR/TGR5 signaling [10,11,41], and gut dysbiosis that perpetuates both through reduced secondary bile acid generation and endotoxin translocation [65,67,68]. Critically, the convergence of a contracted bile acid pool, a shift toward 12α-hydroxylated species, and impaired enterohepatic recycling collectively transforms a mere metabolic surplus of urate into a state of amplified inflammatory vulnerability [13,14]. This integrated perspective not only resolves clinical paradoxes—such as why only a subset of hyperuricemic individuals develop gout [2,3]—but also exposes the mechanistic rationale for intervening at the bile acid–immune interface rather than targeting urate alone [1,4,15].

### 8.2. Emerging Methodologies and Integrative Approaches for Future Research

Despite the conceptual advances synthesized here, the path from mechanistic insight to clinical application remains obstructed by several critical evidence gaps [14,15]. A key gap that warrants emphasis is that many of the mechanistic insights presented in this review, especially those concerning FXR/TGR5-NLRP3 signaling, PPAR-α/XOD regulation, and microbial–bile acid interactions, are derived from preclinical models of other metabolic and inflammatory diseases, including NAFLD, cholestasis, sepsis, and colitis. These conditions have bile acid environments that differ markedly from that of gout, where the total bile acid pool is reduced and hydrophobic secondary bile acids are depleted. Therefore, extrapolation of these mechanisms to gout pathology should be approached with caution, and there is an urgent need for gout-specific mechanistic studies using MSU crystal-induced arthritis models and patient-derived samples to validate the framework proposed here. We identify three overarching priorities that must guide future investigations:

First, establishing causality and temporal dynamics in humans. The vast majority of current data derive from cross-sectional metabolomic snapshots and rodent models that fail to recapitulate spontaneous MSU crystal deposition and recurrent arthritis [8,9,13]. A key priority is the deployment of large-scale, prospective longitudinal cohorts that track bile acid profiles, microbial metagenomics, and inflammatory biomarkers across the disease continuum—from asymptomatic hyperuricemia through acute flares to intercritical periods [1,4,9]. Such designs, complemented by Mendelian randomization studies using genetic instruments for bile acid synthesis enzymes, are essential to disentangle whether observed bile acid alterations are causal drivers or merely downstream epiphenomena of systemic inflammation [12,13,16].

Second, reconciling context-dependent receptor pharmacology with therapeutic safety. Particular attention should be given to the inherent duality of FXR signaling: activation suppresses NLRP3 transcription but simultaneously contracts the bile acid pool, weakening TGR5 agonism [28,38,43]. This therapeutic dilemma calls for mechanism-informed drug design rather than empirical screening [15,43]. The most promising avenues include: (a) gut-restricted TGR5 agonists that avoid on-target cardiovascular and gallbladder side effects [41,48]; (b) selective FXR modulators that bias signaling toward anti-inflammatory pathways while sparing CYP7A1 suppression [45,47]; and (c) dual-target molecules (e.g., URAT1/FXR or FXR/TGR5 hybrids) that decouple urate-lowering from metabolic trade-offs [12,14,76]. Rigorous evaluation of these candidates in gout-specific MSU-induced arthritis models, followed by phase 2 trials with stratified patient enrollment, remains the critical bottleneck for clinical translation [11,29].

Third, moving from empirical microbiota manipulation to defined, standardized interventions. While probiotics and fecal microbiota transplantation hold theoretical appeal, their clinical utility is currently undermined by strain-specific heterogeneity, undefined active metabolites, and lack of potency standardization [25,72,75]. We advocate for a shift toward defined bacterial consortia with characterized bile salt hydrolase and 7α-dehydroxylase activities, or even engineered microbial enzymes, to achieve reproducible reconstitution of the secondary bile acid pool [26,81,90]. Concurrently, the integration of multi-omics profiling (metagenomics, metabolomics, host transcriptomics) into clinical trial frameworks will enable endotype-driven patient stratification—identifying those with a “low-TGR5-signaling” or “12α-OH-predominant” phenotype who are most likely to respond to specific bile acid–targeted regimens [8,9,14,16].

Finally, the translation of natural products—notably diosgenin, berberine, and ursodeoxycholic acid—must advance beyond observational associations into randomized controlled trials with pharmacokinetic optimization (e.g., nanoparticle formulations) to overcome their notoriously low bioavailability [13,29,53,80]. Addressing these interconnected priorities will collectively transform the current preclinical promise into actionable, personalized therapeutic strategies for gout and hyperuricemia [14,16,80]. Ultimately, the most critical unresolved question is whether the observed bile acid alterations drive gout pathogenesis or merely reflect the systemic inflammatory and metabolic disturbances that accompany the disease. This distinction has profound translational implications. If these alterations are causally involved, then restoring bile acid homeostasis—whether through FXR/TGR5 modulation, microbiota-based interventions, or bile acid replacement—could represent a disease-modifying strategy. If they are largely reactive, however, then bile acid metabolites may still hold value as biomarkers for disease activity or as adjunctive targets to mitigate inflammation, but they would not address the root drivers of hyperuricemia and crystal deposition. Resolving this question will require prospective cohort studies with pre-diagnostic metabolomic profiling, Mendelian randomization using genetic instruments for bile acid synthesis enzymes, and gout-specific preclinical models that recapitulate spontaneous MSU crystal deposition and recurrent arthritis. Until such evidence emerges, the targeting of bile acid signaling in gout remains a promising but unproven therapeutic frontier—one that demands gout-specific mechanistic studies and biomarker-qualified clinical trials to resolve causality before clinical adoption.

## Figures and Tables

**Table 1:** Key enzymes, transporters, and receptors in bile acid metabolism and gout pathogenesis.

Component	Type	Primary Function	Specific Role in Gout/HUA	References
CYP7A1	Enzyme (rate-limiting)	Converts cholesterol to 7α-hydroxycholesterol; initiates classical bile acid synthesis	Downregulated by FXR activation; upregulated by diosgenin (FXR antagonist) → reduces uric acid via PPAR-α/XOD	[19,41]
CYP8B1	Enzyme	Determines cholic acid (CA) synthesis; introduces 12α-OH group	12α-OH bile acids (CA, DCA) are elevated in hyperuricemia; linked to XOD activity	[20,21]
CYP27A1	Enzyme	Initiates alternative (acidic) pathway; produces CDCA	↑ in hyperuricemic liver; generates CDCA (a PPAR-α ligand) which may indirectly upregulate XOD when alternative pathway is overactivated.	[18,22]
Bile salt hydrolase (BSH)	Bacterial enzyme	Deconjugates bile acids (TCA, GCA → CA, CDCA)	Reduced in gout dysbiosis; probiotic BSH restores FXR/TGR5 ligands	[25]
7α-dehydroxylase	Bacterial enzyme	Converts CA → DCA, CDCA → LCA	Decreased in gout → reduced DCA/LCA → impaired TGR5 signaling	[25,26]
ASBT	Transporter (ileal)	Reabsorbs bile acids from ileum	Impaired in hyperuricemia → enterohepatic stagnation → conjugated bile acid accumulation	[32,33]
NTCP	Transporter (hepatic)	Takes up bile acids from portal blood	Downregulated in hyperuricemia → impaired hepatic uptake of conjugated bile acids → elevated systemic BA load → competes with urate for renal OAT1/3 excretion.	[30,32]
FXR	Nuclear receptor	Regulates bile acid synthesis (SHP, FGF19); suppresses NLRP3 transcription	Context-dependent: protective in low-BA states (gout); problematic in cholestasis	[5,39,40]
TGR5	GPCR	Activates cAMP/PKA; inhibits NLRP3 assembly; stimulates GLP-1	Reduced DCA/LCA → impaired TGR5 → decreased GLP-1 → increased IL-1β	[6,37,44]
PPAR-α	Nuclear receptor	Regulates fatty acid oxidation; upregulates XOD	Activated by hydrophobic bile acids → increases uric acid production	[49,50]
XOD	Enzyme	Catalyzes hypoxanthine → xanthine → uric acid	Target of allopurinol/febuxostat; upregulated by PPAR-α	[2,50]
OAT1/OAT3	Transporters (renal)	Uptake of organic anions (urate, bile acids)	Conjugated bile acids (e.g., GCDCA) accumulate in hyperuricemia and competitively inhibit OAT1/3-mediated renal urate uptake → reduced uric acid excretion (a “second hit” for hyperuricemia).	[53]

Footnote: The upward arrow symbol ↑ is used throughout this text to represent significantly elevated concentration, gene expression, or protein abundance of the corresponding molecule/gene.

**Table 2:** Molecular mechanisms linking bile acid dysregulation to PPAR-α activation.

Category	Mechanism/Basis	Key Evidence	Ref.
Why hydrophobic bile acids (CDCA, DCA, LCA)?	Greater lipophilicity (LCA > DCA > CDCA > CA) facilitates membrane partitioning and intracellular accumulation, enabling concentrations sufficient for receptor modulation	Physicochemical property; intracellular accumulation correlates with hydrophobicity	[15]
FXR-dependent pathway	Human PPARA promoter contains a functional FXR response element (FXRE); FXR/RXR binds and activates transcription	CDCA and GW4064 induce PPARA mRNA in HepG2 cells; mutation of FXRE abolishes bile acid response; 3-fold promoter activation with FXR/RXR + CDCA	[29]
FXR-independent pathway (activation)	Bile acids—particularly LCA—directly or indirectly activate PPAR-α via membrane receptors (TGR5/cAMP), ROS-MAPK, and PKC pathways	LCA activates PPAR-α in FXR-null mice; TGR5 agonists increase PPAR-α target genes	[15,56]
FXR-independent pathway (antagonism)	Bile acids impair recruitment of transcriptional coactivators to PPAR-α, antagonizing its transactivation	Bile acid-enriched diet reduces Wy-14,643-induced hepatomegaly and target gene expression	[55]
Species-specific regulation	FXRE is conserved in human but not murine PPARA promoter	Murine promoter lacks FXRE and is unresponsive to bile acids	[29]
Context-dependent net effect	Bile acids can act as agonists (via FXR-mediated transcription) or antagonists (via coactivator interference), depending on cell type, concentration, and metabolic state	Dual effects observed in different experimental systems; potential implications in cholestasis and metabolic diseases	[55]

**Table 3:** Distinction between human-validated mechanisms and preclinical hypotheses in bile acid–gout research.

Mechanism	Evidence in Human Samples	Evidence in Preclinical Models	Validation Status
Bile acid pathway alterations in gout	Multiple metabolomic studies-37-40-38	Animal models	Human-validated
Gut microbiota–bile acid axis–hyperuricemia association	Xiangya Study, prospective cohort	Animal models	Human-validated(association)
FXR expression and function	Human tissues and cell lines [44]	Extensive	Human-validated
FXR–NLRP3 transcriptional suppression	No direct human gout data	Murine macrophages, animal models-37	Preclinical
TGR5–cAMP/PKA–NLRP3 inhibition	No gout patient data; TGR5 agonists in early clinical development for other diseases-	Rodent macrophages, cell lines [10]	Preclinical
PPAR-α/XOD–urate overproduction	No direct human evidence	Rodent models, hepatocyte lines [54]	Preclinical
12α-OH bile acids as causal mediators	No human causal evidence	Rat model-55	Preclinical

**Table 4:** Summary of gut bacterial taxa consistently reported as altered in gout patients, with functional roles and associated bile acid metabolic implications.

Functional Category	Taxon	Change in Gout	Functional Role in Gut Ecosystem	Bile Acid–Related Implication
SCFA producers	*Faecalibacterium prausnitzii*	↓	Butyrate production; anti-inflammatory; IL-10 induction	Butyrate deficiency → impaired HDAC inhibition → disinhibited NLRP3; shares depletion pattern with other metabolic diseases [80,81]
	*Coprococcus*	↓	Butyrate production; SCFA generation	Reduced butyrate → weakened anti-inflammatory effects on NLRP3 and intestinal epithelium [16]
	*Anaerostipes*	↓	Butyrate production; beneficial for host metabolism	Reduced butyrate → impaired TGR5-mediated GLP-1 secretion and epithelial energy supply [82]
	*Lachnospira*	↓	Butyrate production; polysaccharide fermentation	Depleted during acute gout flares → impaired SCFA-mediated inflammation resolution [16]
	*Ruminococcus*	↓	Polysaccharide degradation; SCFA production	Decreased during flares; causal association with gout via Mendelian randomization [12]
	*Dialister*	↓	SCFA production	Further decreased during flares → reduced SCFA-mediated protection [16]
	*Christensenellaceae* R-7 group	↓	Anti-inflammatory; metabolic homeostasis	Reduced abundance correlates with impaired metabolic and immune homeostasis [83]
	*Enterococcus*	↓	Butyrate production; SCFA generation	Reduced SCFAs → weakened gut barrier and immune regulation [82]
BSH-active/bile acid–modifying	*Bifidobacterium*	↓	Bile salt hydrolase (BSH) activity; probiotic; anti-inflammatory	Reduced BSH → decreased deconjugation → impaired generation of FXR/TGR5 ligands; directly impacts secondary bile acid pool [76]
	*Clostridium* (clusters XIVa & XI)	↓ diversity	7α-dehydroxylation (CA → DCA, CDCA → LCA); butyrate production	Directly reduces DCA/LCA generation → impaired TGR5 signaling and antimicrobial barrier function [25,26]
	*Lactobacillus*	↓ (in multiple studies)	BSH activity; probiotic; anti-inflammatory	Reduced BSH → impaired deconjugation and secondary bile acid generation [76,84]
Pro-inflammatory opportunists	*Prevotella*	↑	Polysaccharide degradation; associated with pro-inflammatory milieu	May modulate bile acid composition via altered gut microenvironment; enriched in hyperuricemia [83]
	*Fusobacterium*	↑	Pro-inflammatory; opportunistic pathogen	Promotes systemic inflammation that may suppress FXR/TGR5 expression; enriched in hyperuricemia [12,83]
	*Bacteroides* (certain species)	↑ (species-specific)	Complex polysaccharide metabolism; enzyme modulation in purine metabolism	Alters bile acid deconjugation and secondary bile acid generation; B. caccae and B. xylanisolvens enriched in gout [83]
	*Bilophila*	↑	Sulfate-reducing; pro-inflammatory; bile acid-tolerant	Expansion reflects altered bile acid pool hydrophobicity; its bile acid tolerance permits overgrowth when antimicrobial DCA/LCA are reduced [79]
	*Alistipes*	↑	Associated with purine metabolism enzyme modulation	May indirectly affect bile acid enterohepatic circulation; enriched in gout [83]
	*Phascolarctobacterium*	↑	Modulates enzymatic activity in purine metabolism	Associated with altered bile acid pool composition in metabolic disorders [85]
Other	*Collinsella*	↓	Anti-inflammatory properties	Reduced abundance associated with metabolic dysfunction; may affect lipid and bile acid metabolism [83]
	*Enterobacteriaceae*	↓ (in gout vs. controls)	Amino acid metabolism; environmental sensing; urate degradation	Reduced amino acid metabolism → increased serum uric acid and CRP; functional implications less clear than other taxa [83]

Note: ↑ = increased abundance in gout/hyperuricemia vs. healthy controls; ↓ = decreased abundance.

**Table 5:** Summary of bile acid profile alterations in gout and hyperuricemia.

Parameter	Change Observed	Proposed Mechanism/Implication	References
Total bile acid pool	↓ (most studies)	Impaired hepatic synthesis (CYP7A1?), reduced intestinal reabsorption (ASBT dysfunction), systemic inflammation	[8,13,28]
Primary bile acids (CA)	↑ (in liver/serum)	Compensatory increase in de novo synthesis; associated with 12α-OH elevation	[13,19]
Secondary bile acids (DCA, LCA)	↓ (serum/fecal)	Gut dysbiosis: loss of 7α-dehydroxylase-producing *Clostridium* spp.	[13,25]
Conjugated bile acids (TCA, GCA, TDCA)	↑ (ileal content)	Impaired enterohepatic circulation; ASBT dysfunction	[13,32]
12α-hydroxy bile acids	↑	Linked to CYP8B1 activity; correlates with XOD-mediated urate production; biomarker for disease severity	[13,21,50]
Hydrophobicity index	↓	Reduced DCA/LCA leads to weaker TGR5 activation; less antimicrobial activity	[7,37]
UDCA (in NAFLD patients)	↓ or unchanged	Supplementation lowers urate; possible XOD inhibition	[13,79]

Note: Direction of changes may vary across studies; heterogeneity arises from differences in study design, sample types, and patient populations. ↑ = increased levels; ↓ = decreased levels; ↓ or unchanged = reduced or unaltered levels.

**Table 6:** Therapeutic strategies targeting bile acid pathways in gout: mechanisms, evidence, and limitations.

Strategy Category	Representative Agents	Primary Target/Mechanism	Key Preclinical/Clinical Evidence in Gout/HUA	Major Limitations & Safety Concerns
I. Nuclear Receptor Modulators	OCA, GW4064 (Agonists); Diosgenin (Antagonist); Benzbromarone derivatives (Dual URAT1/FXR)	FXR: Agonists suppress NLRP3 transcription; Antagonists ↑ CYP7A1 (↑ BA pool)	Agonists reduce MSU-induced IL-1β in murine models; Diosgenin lowers urate in rats	Context-dependent: Agonists reduce TGR5 ligands (DCA/LCA); Antagonists lose anti-inflammatory brake. Pruritus & LDL ↑ in clinical trials.
II. Membrane Receptor (GPCR) Agonists	INT-777, Oleanolic acid, SB756050	TGR5: Activates cAMP/PKA → blocks NLRP3 assembly; ↑ GLP-1 (systemic anti-inflammation)	Suppresses IL-1β release in MSU-stimulated macrophages; Improves metabolic inflammation	On-target toxicity: Risk of tachycardia & gallbladder stasis. No gout-specific RCTs completed yet.
III. Microbiome & Barrier Restorers	Probiotics (*Lactobacillus*, *Bifidobacterium*), FMT, Prebiotics	Restores BSH/7α-dehydroxylase → ↑ DCA/LCA (TGR5 ligands); repairs gut barrier → ↓ LPS translocation	Probiotics reshape BA profiles and lower XOD activity in hyperuricemic rats Human RCT ongoing.	Strain-specific effects; FMT lacks standardization; long-term safety unknown.
IV. Bile Acid Replacement/Multi-target	Ursodeoxycholic acid (UDCA), Berberine, Resveratrol	UDCA: ↑ hydrophilicity (pool remodeling), possible XOD inhibition; Berberine: activates TGR5 & modulates microbiota	UDCA reduced serum urate in NAFLD patients (from 386 to 334 μmol/L). Berberine shows anti-inflammatory effects.	Low bioavailability (natural products); UDCA mechanism remains unclear; efficacy in gout without NAFLD unproven.

Footnote: ↑ = increased levels; ↓ = decreased levels.

**Table 7:** Clinical development status, safety profile, and regulatory considerations of bile acid–targeted therapies.

Agent	Target	Highest Development Phase (Indication)	Regulatory Status	Dosing	Key Safety Concerns	Relevance to Gout
Obeticholic acid (OCA, INT-747)	FXR agonist	Approved (PBC); Phase 3 (NASH)	FDA-approved 2016 for PBC-	5–10 mg/day; adjust in hepatic impairment	Pruritus (37.8%), fatigue, nausea, LDL ↑, HDL ↓, hepatic complications in cirrhosis-	Anti-inflammatory potential; no gout-specific trials; safety concerns may limit use
INT-787	FXR agonist	Phase 1 (healthy volunteers)-	Investigational	Not established	Under evaluation in phase 1	Novel hydrophilic FXR agonist; early-stage
Cilofexor (GS-9674)	FXR agonist	Phase 2 (NASH)-3	Investigational	Not established	Improved safety profile vs. OCA	Non-bile-acid FXR agonist; lower pruritus risk
Tropifexor	FXR agonist	Phase 2 (NASH)-	Investigational	Not established	Under evaluation	Second-generation FXR agonist
MET409	FXR agonist	Phase 1b (NASH)-	Investigational	Not established	Lower pruritus and LDL-c increment vs. OCA	Improved therapeutic window
HPG1860	FXR agonist (non-bile-acid)	Phase 2a (NASH)-3	Investigational	Not established	Under evaluation	Non-bile-acid scaffold; may avoid some bile-acid-related side effects
SB756050	TGR5 agonist	Phase 2 (T2DM)—discontinued-24	Discontinued	N/A	Failed to show expected PD profile	Development halted
BAR502	Dual FXR/TGR5 agonist	Early-phase clinical-24	Investigational	Not established	Under evaluation	Dual targeting may offer synergistic benefits
Gut-restricted TGR5 agonists	TGR5 agonist (intestinal)	Preclinical/early clinical-	Investigational	Not established	Reduced systemic side effects (gallbladder, cardiovascular)	Promising approach to avoid on-target toxicity
UDCA	Bile acid (hydrophilic)	Approved (PBC, gallstones)	FDA-approved; well-established safety	10–15 mg/kg/day	Minimal; generally well-tolerated-58	Reduces uric acid in NAFLD patients-58; needs gout-specific trials
Probio-X + febuxostat	Microbiota modulation	Phase 2 (gout)-	Investigational	Not established	Under evaluation	First probiotic RCT in gout; bile acid endpoints not primary

Footnote: ↑ = increased levels; ↓ = decreased levels.

## Data Availability

No new data were created or analyzed in this study.

## Abbreviations

The following abbreviations are used in this manuscript:

7α-dehydroxylase	7α-dehydroxylase (bacterial enzyme)
AhR	Aryl hydrocarbon receptor
ASBT	Apical sodium-dependent bile acid transporter
AST	Aspartate aminotransferase
ALT	Alanine aminotransferase
BAAT	Bile acid-CoA:amino acid N-acyltransferase
BSH	Bile salt hydrolase
CA	Cholic acid
cAMP	Cyclic adenosine monophosphate
CDCA	Chenodeoxycholic acid
CYP7A1	Cholesterol 7α-hydroxylase
CYP8B1	Sterol 12α-hydroxylase
CYP27A1	Sterol 27-hydroxylase
DAMP	Damage-associated molecular pattern
DCA	Deoxycholic acid
FGF19	Fibroblast growth factor 19
FMT	Fecal microbiota transplantation
FXR	Farnesoid X receptor
GCA	Glycocholic acid
GLP-1	Glucagon-like peptide-1
GLP-1R	Glucagon-like peptide-1 receptor
GPR43	G-protein coupled receptor 43
GPR109a	G-protein coupled receptor 109a
HDAC	Histone deacetylase
IL-1β	Interleukin-1 beta
IL-6	Interleukin-6
IL-18	Interleukin-18
IRS-2	Insulin receptor substrate-2
LCA	Lithocholic acid
LPS	Lipopolysaccharide
MAMP	Microbial-associated molecular pattern
MAPK/ERK	Mitogen-activated protein kinase/extracellular signal-regulated kinase
MSU	Monosodium urate
NAFLD	Non-alcoholic fatty liver disease
NF-κB	Nuclear factor kappa-light-chain-enhancer of activated B cells
NLRP3	LRR and PYD domains-containing protein 3
NTCP	Sodium–taurocholate cotransporting polypeptide
OAT1	Organic anion transporter 1
OAT3	Organic anion transporter 3
PAMP	Pathogen-associated molecular pattern
PBC	Primary biliary cholangitis
PKA	Protein kinase A
PPAR-α	Peroxisome proliferator-activated receptor α
PPRE	Peroxisome proliferator response element
PXR	Pregnane X receptor
ROS	Reactive oxygen species
RORγt	Retinoid-related orphan receptor γt
SCFA	Short-chain fatty acid
SHP	Small heterodimer partner
SIRT1	Sirtuin 1
TCA	Taurocholic acid
TDCA	Taurodeoxycholic acid
TGR5	G protein-coupled bile acid receptor 1
TLR4	Toll-like receptor 4
TNF-α	Tumor necrosis factor alpha
UDCA	Ursodeoxycholic acid
URAT1	Urate transporter 1
VDR	Vitamin D receptor
XOD	Xanthine oxidase
